# Epigenetic suppression of human telomerase (*hTERT*) is mediated by the metastasis suppressor NME2 in a G-quadruplex–dependent fashion

**DOI:** 10.1074/jbc.M117.792077

**Published:** 2017-07-17

**Authors:** Dhurjhoti Saha, Ankita Singh, Tabish Hussain, Vivek Srivastava, Suman Sengupta, Anirban Kar, Parashar Dhapola, Vishnu Dhople, Ramesh Ummanni, Shantanu Chowdhury

**Affiliations:** From the ‡ Genomics and Molecular Medicine Unit,; § G.N.R. Knowledge Centre for Genome Informatics, and; ‖ Academy of Scientific & Innovative Research (AcSIR), CSIR-Institute of Genomics and Integrative Biology, Council of Scientific and Industrial Research (CSIR), Mathura Road, New Delhi 110025, India and; ¶ Centre for Chemical Biology, CSIR-Indian Institute of Chemical Technology, Hyderabad 500007, India

**Keywords:** chromatin modification, epigenetics, G-quadruplex, telomerase reverse transcriptase (TERT), transcription regulation, NME2, REST repressor complex

## Abstract

Transcriptional activation of the human telomerase reverse transcriptase (*hTERT*) gene, which remains repressed in adult somatic cells, is critical during tumorigenesis. Several transcription factors and the epigenetic state of the *hTERT* promoter are known to be important for tight control of *hTERT* in normal tissues, but the molecular mechanisms leading to *hTERT* reactivation in cancer are not well-understood. Surprisingly, here we found occupancy of the metastasis suppressor non-metastatic 2 (NME2) within the *hTERT* core promoter in HT1080 fibrosarcoma cells and HCT116 colon cancer cells and NME2-mediated transcriptional repression of *hTERT* in these cells. We also report that loss of NME2 results in up-regulated *hTERT* expression. Mechanistically, additional results indicated that the RE1-silencing transcription factor (REST)–lysine-specific histone demethylase 1 (LSD1) co-repressor complex associates with the *hTERT* promoter in an NME2-dependent way and that this assembly is required for maintaining repressive chromatin at the *hTERT* promoter. Interestingly, a G-quadruplex motif at the *hTERT* promoter was essential for occupancy of NME2 and the REST repressor complex on the *hTERT* promoter. In light of this mechanistic insight, we studied the effects of G-quadruplex–binding ligands on *hTERT* expression and observed that several of these ligands repressed *hTERT* expression. Together, our results support a mechanism of *hTERT* epigenetic control involving a G-quadruplex promoter motif, which potentially can be targeted by tailored small molecules.

## Introduction

Specialized DNA–protein assemblies called telomeres protect chromosome ends from being detected as DNA breaks (1, 2). The ribonucleoprotein telomerase adds *de novo* repeats at the end of telomeres to maintain telomere length (3). Human telomerase comprises the catalytic reverse transcriptase (hTERT)^7^ and an RNA component (hTR) that provides the template for addition of telomeric repeats (4, 5). Lack of telomerase results in shortening of telomeres because of the end replication problem (6), and cells with critically short telomeres activate the DNA damage response, leading to cell cycle arrest or apoptosis (7, 8). This is the case in most normal somatic cells, which lack telomerase. Most cancer cells, however, have high levels of telomerase, and telomere length is maintained for initiation and survival of tumors (9). In normal cells, the limiting factor for telomerase activity is the level of *hTERT* mRNA, which is under strong transcriptional control (10). In contrast, in about 85% of all cancers, *hTERT* expression is reactivated (11), leading to malignant transformation and aggressive metastasis in many cases (12). The molecular mechanisms that underlie *hTERT* reactivation from otherwise tight transcriptional control in normal somatic cells remain poorly understood.

In this context, the metastasis suppressor non-metastatic 2 (NME2; also known as nm23-H2) is of interest (13). Human *nm23* has several isoforms; of these, H1 (or NME1) and H2 are the most studied (14–16). The role of NME2 in metastases suppression is well-described: overexpression of NME2 results in reduced metastasis of human oral squamous carcinoma, breast carcinoma, and murine melanoma cells (17–19), and the level of *NME2* expression negatively correlates with advanced/metastatic stages in several tumor types (20). Notably, independent studies reported NME2-mediated transcription regulation of c-*myc* where association of NME2 to a G-rich sequence motif within the nuclease-hypersensitive element of the c-*myc* promoter was revealed (21). NME2 was also reported to regulate *PDGF-A* and vinculin transcriptionally, supporting its role as a regulatory factor (22, 23).

Herein we show that transcription of *hTERT* remains repressed in the presence of NME2, and loss of NME2 results in up-regulation of *hTERT* expression. NME2 binds to the *hTERT* core promoter, and the REST repressor complex associates with the *hTERT* promoter in an NME2-dependent manner. Results also revealed that the presence of an intact G-rich DNA secondary structure G-quadruplex (G4) motif in the *hTERT* core promoter was required for association of NME2 and the REST repressor complex at the *hTERT* promoter. Notably, in the presence of NME2 and the REST repressor complex, epigenetic alterations restricted permissiveness of the *hTERT* promoter. Because altered NME2 has been detected in multiple cancer tissues (14, 17–19), it is of interest to understand the mechanisms underlying low NME2 and enhanced *hTERT* expression/activation.

## Results

### NME2 associates with the hTERT core promoter and transcriptionally represses hTERT

**Figure 1.**
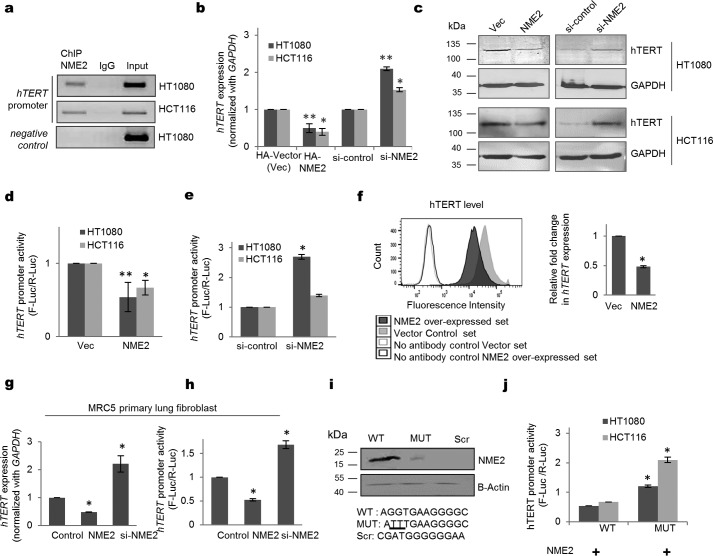
**NME2 occupies the *hTERT* promoter and reduces its expression.** *a*, NME2 ChIP showing occupancy of NME2 on the *hTERT* promoter in HT1080 and HCT116 cell lines. *ptprc* gene serves as a negative control. *b* and *c*, real-time PCR (*b*) and Western blot analysis (*c*) for *hTERT* expression (49, 50) upon NME2 overexpression and NME2 silencing in HT1080 and HCT116 cells. The *top panels* show *hTERT* expression, and the *lower panels* show the expression of the *GAPDH* loading control (*c*). *Error bars* represent S.E. (three biological replicates); * indicates a *p* value <0.05; ** indicates a *p* value <0.005. *d* and *e*, luciferase reporter assay to measure *hTERT* promoter activity upon NME2-overexpressed (*d*) and -silenced conditions (*e*). *F-luc*, firefly luciferase; *R-luc*, *Renilla* luciferase. *Error bars* represent S.E. (three biological replicates); * indicates a *p* value <0.05; ** indicates a *p* value <0.005. *f*, flow cytometry analysis shows reduced expression of *hTERT* in GFP-NME2 stably overexpressing cells in comparison with GFP vector (*Vec*) HT1080 cells. *Error bars* represent S.E. (three biological replicates); * indicates a *p* value <0.05. *g* and *h*, *hTERT* expression (*g*) and promoter activity (*h*) were measured in primary MRC5 normal lung fibroblasts in NME2-overexpressed and -silenced conditions. *Error bars* represent S.E. (three biological replicates); * indicates a *p* value <0.05. *i*, oligo pulldown assay for NME2 using biotin-labeled oligos with WT, MUT, and scrambled (*Scr*) NME2 motifs. The *lower panel* represents the loading control. *j*, luciferase reporter assay for *hTERT* promoter activity after overexpressing NME2 along with wild-type and mutant NME2 motifs present in *hTERT* promoter construct. *Error bars* represent S.E. (three biological replicates); * indicates a *p* value <0.05.

We noted a putative NME2-binding site on the *hTERT* core promoter based on a previously reported motif from NME2 chromatin immunoprecipitation (ChIP)-sequencing experiments (24). Here we performed ChIP-PCR, with primers (spanning from +40 to −230 bp with respect to the *hTERT* transcription start site) flanking the putative NME2-binding site, first in HT1080 fibrosarcoma cells and then in HCT116 colon cancer cells to confirm NME2 occupancy at the *hTERT* promoter (Fig. 1*a* and supplemental Fig. S1a). To test the functional significance of the NME2 occupancy, endogenous *hTERT* expression was checked in NME2-overexpressed or -silenced conditions in HT1080 and HCT116 cells. We found clear repression and an increase in *hTERT* expression upon NME2 overexpression or silencing, respectively, and similar changes in hTERT protein levels (Fig. 1, *b* and *c*, and supplemental Fig. S1b). The core promoter of *hTERT* was cloned into a luciferase reporter, and promoter activity was measured under NME2-altered conditions in HT1080 and HCT116 cells. NME2 expression and *hTERT* promoter activity were found to be inversely correlated (Fig. 1, *d* and *e*). Together, these data suggested NME2-mediated transcriptional repression of *hTERT* in cancer cells.

This was further supported by fluorescence-activated cell sorting (FACS) and immunofluorescence analysis using stable NME2-overexpressing cells, which showed reduced expression of *hTERT* relative to vector-transformed cells (Fig. 1*f* and supplemental Fig. S1, c and d). Telomerase enzymatic activity was measured under NME2-overexpressed or -silenced conditions using a telomere repeat amplification protocol, and a reduction or an increase in telomerase activity, respectively, was observed (supplemental Fig. S1e). Based on this, we asked whether NME2 repressed *hTERT* in normal primary cells. In primary lung fibroblast MRC5 cells, we found repression or enhanced *hTERT* expression and promoter activity under NME2-overexpressed or -silenced conditions, respectively (Fig. 1, *g* and *h*).

To validate the occupancy of NME2 on the *hTERT* promoter, an oligonucleotide pulldown assay was performed. A putative 12-mer (−115 to −127 bp from *hTERT* transcription start site) representing the NME2 DNA-binding motif (23) or its mutated/scrambled negative controls were used. Pulldown using anti-NME2 antibody showed specific binding to the unaltered (wild-type (WT)) relative to mutated (MUT) or scrambled motifs (Fig. 1*i*). Furthermore, a reporter plasmid in which the mutant NME2 motif was inserted did not suppress promoter activity in the presence of NME2 in HT1080 and HCT116 cells (Fig. 1*j*). Together, these results suggested that NME2 binding was required for transcriptional repression of *hTERT.*

Next, we checked the role, if any, of NME1, a close homolog of NME2, in transcriptional repression on *hTERT. hTERT* expression and protein levels remained unaltered when NME1 was specifically silenced (supplemental Fig. S1, f and g); *hTERT* promoter activity also was not affected upon NME1 silencing in HT1080 cells (supplemental Fig. S1h).

### NME2 maintains repressive chromatin at the hTERT promoter by engaging the REST–LSD1 repressor complex

**Figure 2.**
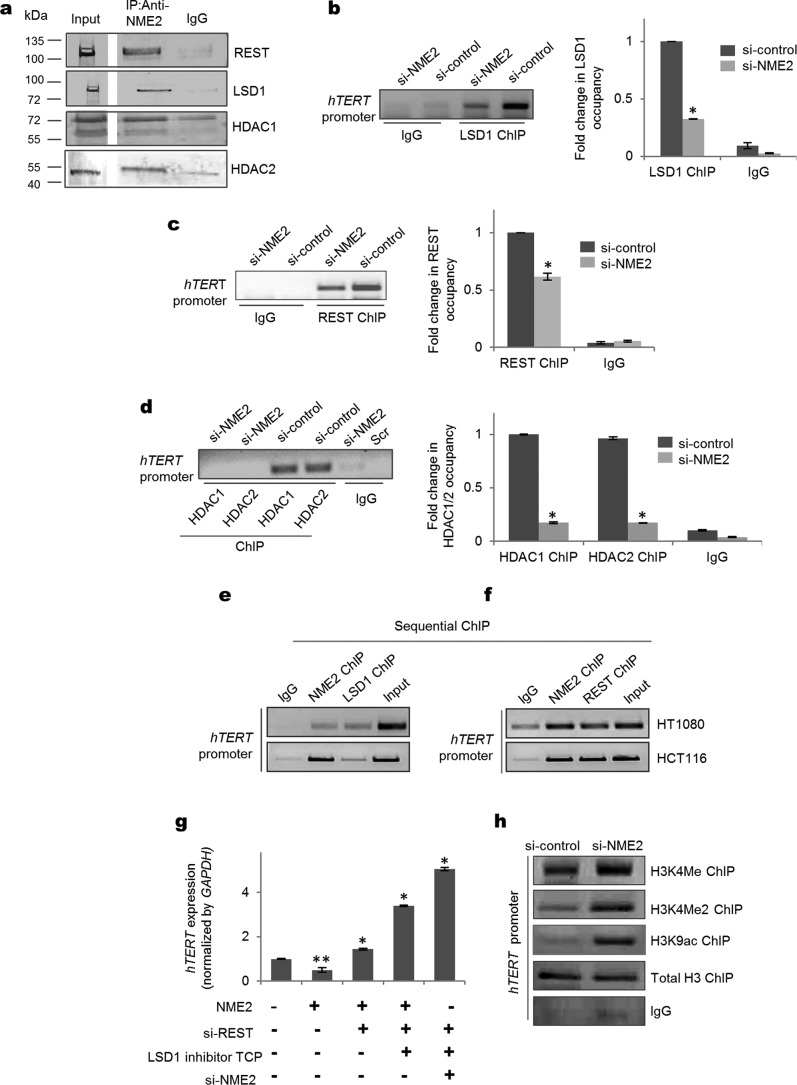
**NME2 interacts with REST complex and alters chromatin marks on *hTERT* to repress transcription.** *a*, co-IP assay for validation of REST complex component interaction with NME2 as obtained from LC-MS/MS results. Co-IP for REST, LSD1, HDAC1, and HDAC2 was performed using nuclear extract from HT1080 cells. *b–d*, LSD1 (*b*), REST (*c*), and HDAC1/2 (*d*) ChIP in the NME2 transiently silenced condition showing reduced occupancy on the *hTERT* promoter. The histogram represents the -fold change in LSD1, REST, and HDAC1/2 occupancy on the *hTERT* promoter. *Error bars* represent S.E. (three biological replicates); * indicates a *p* value <0.05. *e* and *f*, sequential chromatin immunoprecipitation for NME2–LSD1 (*e*) and NME2–REST (*f*) followed by PCR of *hTERT* promoter in HT1080 and HCT116 cell lines. *g*, *hTERT* mRNA expression was measured after silencing of REST and NME2, blocking of LSD1 using TCP, and combinations of both treatments. *Error bars* represent S.E. (three biological replicates); * indicates a *p* value <0.05; ** indicates a *p* value <0.005. *h*, ChIP for active histone methylation (H3K4me and H3K4me2) and acetylation (H3K9ac) marks and total histone3 (H3) on *hTERT* promoter in NME2-silenced conditions in HT1080 cells. *Scr*, scrambled.

To understand the mechanism underlying NME2-mediated *hTERT* repression, we asked whether other binding partners of NME2 were involved. To find possible nuclear interacting partners of NME2, we used LC-MS/MS after immunoprecipitation with anti-NME2 antibody from nuclear extract of HT1080 cells. Using a stringent analysis cutoff of 1% false discovery rate, 120 interacting partners of NME2 were detected (supplemental Fig. S2a and Table S1) that belonged to several important classes including chromatin remodeling (Gene Ontology analysis; supplemental Fig. S2b). Of these, to specifically understand possible mechanisms underlying NME2-induced *hTERT* repression, we focused on chromatin modifiers HDAC1 and HDAC2. Interaction of NME2 with both HDAC1 and HDAC2 was further confirmed using co-immunoprecipitation (co-IP) experiments (Fig. 2*a*).

HDAC1 and HDAC2 are integral members of the REST repressor complex that engages the lysine-specific demethylase LSD-1 (25, 26). Therefore, we checked whether NME2 interacts with REST and LSD1. Co-IP with anti-NME2 antibody showed clear enrichment of REST and LSD1 in the anti-NME2-immunoprecipitated fraction compared with its isotype control IgG (Fig. 2*a*). In addition, reverse co-IP with anti-REST or anti-LSD1 confirmed NME2 interaction with REST–LSD1 (supplemental Fig. S2c).

Next, we checked for REST and LSD1 occupancy on the *hTERT* promoter and determined whether this was NME2-dependent. Both REST and LSD1 ChIPs showed occupancy on the *hTERT* promoter where the ChIP signals were significantly reduced under NME2-silenced conditions (Fig. 2, *b* and *c*, and supplemental Fig. S2d). We also found HDAC1 and HDAC2 occupancy on the *hTERT* promoter to be dependent on NME2 (Fig. 2*d*). Moreover, sequential ChIP for NME2–REST and NME2–LSD1 further supported co-occupancy of NME2–REST–LSD1 complex on the *hTERT* promoter in HT1080 and HCT116 cells (Fig. 2, *e* and *f*).

Next, we asked whether NME2-mediated engagement of REST–LSD1 affected *hTERT* expression. siRNA against REST attenuated NME2-induced repression of *hTERT* expression, which was further derepressed using the LSD1 inhibitor tranylcypromine (TCP) (Fig. 2*g*). Silencing of all three, NME2, REST, and LSD1, led to a further increase in *hTERT* expression (Fig. 2*g*). An increase in *hTERT* expression and promoter activity was also evident upon inhibition of REST and LSD1 independently or together (supplemental Fig. S2, e and f). In addition, histone methylation ChIP results showed an increase in histone activation marks (H3K4me2 and H3K4me) following silencing of REST (supplemental Fig. S2g). Taken together these results show role of NME2 in promoting repressive chromatin at the *hTERT* promoter through the REST–LSD1 complex to suppress *hTERT* expression.

Conversely, we expected permissive chromatin at the *hTERT* promoter upon silencing NME2. We checked histone activation marks H3K9ac, H3K4Me2, and H3K4Me and found that all three marks were clearly enhanced in cells treated with siRNA against NME2 relative to control cells (si-control), whereas there was no significant change in total histone H3 occupancy (Fig. 2*h*).

### NME2 mutants devoid of DNA-binding function are unable to repress hTERT transcription

**Figure 3.**
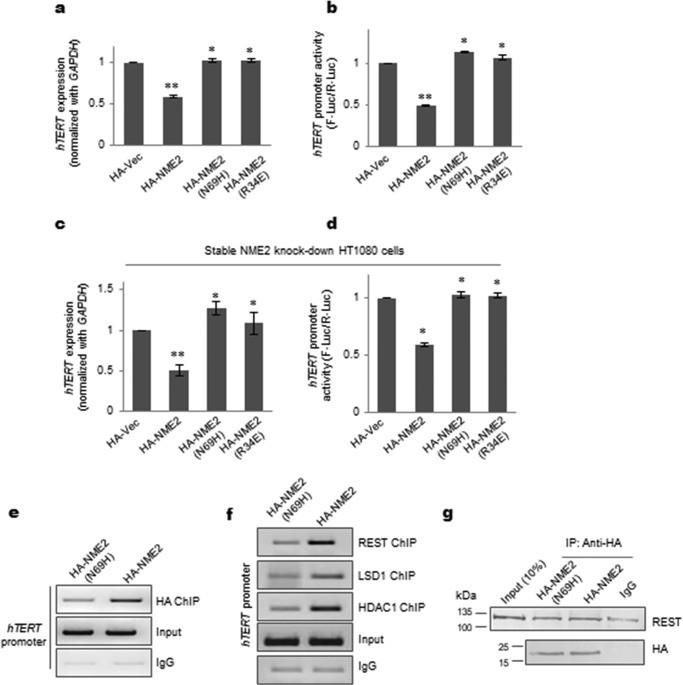
**hTERT transcription repression is dependent on DNA binding by NME2.** *a* and *b*, *hTERT* mRNA (*a*) and promoter activity (*b*) following expression of HA-tagged proteins HA-NME2, HA-NME2(N69H), and HA-NME2(R34A) (R34A and N69H are well-characterized NME2 mutants lacking DNA-binding activity) or the HA vector (*Vec*) control in HT1080 cells; *GAPDH* was used as a control for mRNA expression. *Error bars* represent S.E. (three biological replicates); *, *p* < 0.05; **, *p* < 0.005. *c* and *d*, in a stable NME2-silenced background, *hTERT* mRNA (*c*) and promoter activity (*d*) were checked upon independent overexpression of HA-NME2, HA-NME2(N69H), HA-NME2(R34A), or the HA vector control; *GAPDH* was used as a control for mRNA expression. *Error bars* represent S.E. (three biological replicates); *, *p* < 0.05; **, *p* < 0.005. *e*, occupancy at the *hTERT* promoter was reduced in the case of HA-NME2(N69H) relative to HA-NME2 following ChIP using anti-HA antibody. *f*, occupancy of the components of the REST repressor complex (REST, LSD1, and HDAC1) at the *hTERT* promoter was reduced in cells overexpressing HA-NME2(N69H) relative to cells expressing HA-NME2. *g*, NME2/mutant co-IP with REST. Nuclear lysate prepared from cells expressing either HA-NME2 or HA-NME2(N69H) immunoprecipitated with anti-HA antibody (*top panel*) was probed with anti-REST antibody; total anti-HA was used as a loading control (*lower panel)*.

Mutants of NME2 that are unable to bind DNA (N69H and R34A) were reported earlier (27, 28). Using these mutants, we checked whether DNA binding by NME2 was required for *hTERT* repression. Both the mutants, NME2(N69H) and NME2(R34A), did not show any repression of *hTERT* expression or promoter activity in HT1080 cells (Fig. 3, *a* and *b*). This was further confirmed using stable NME2 knockdown cells (supplemental Fig. S3a). As expected, on stable knockdown of NME2, telomerase expression and promoter activity increased significantly (supplemental Fig. S3, b and c). *hTERT* repression and reduced promoter activity were observed on inducing HA-tagged NME2 (HA-NME2) but not when HA-NME2(N69H) or HA-NME2(R34A) were induced (Fig. 3, *c* and *d*, and supplemental Fig. S3d). Furthermore, using the mutant NME2(N69H), we checked whether occupancy of the REST–LDS1 complex was altered vis-à-vis wild-type NME2. First, using ChIP with HA antibody, we confirmed relatively reduced occupancy of the NME2(N69H) mutant compared with wild-type NME2 on the *hTERT* promoter (Fig. 3*e*). Next, using ChIP, we observed reduced occupancy of REST at the *hTERT* promoter in the case of NME2(N69H) compared with NME2 (Fig. 3*f*) that was not due to any loss of REST interaction with the mutant NME2(N69H) as shown by co-IP experiments in HT1080 cells (Fig. 3*g*). Consistent with reduced REST occupancy, we further found both LSD1 and HDAC1 occupancy to be reduced at the *hTERT* promoter in the case of NME2(N69H) relative to wild-type NME2 (Fig. 3*f*). Together, these studies confirmed DNA binding by NME2 to be a key factor in occupancy of the REST–LSD1 complex at the *hTERT* promoter and *hTERT* repression by NME2.

### G-quadruplex motif at the hTERT promoter was required for NME2 and REST–LSD1 occupancy at the hTERT promoter

**Figure 4.**
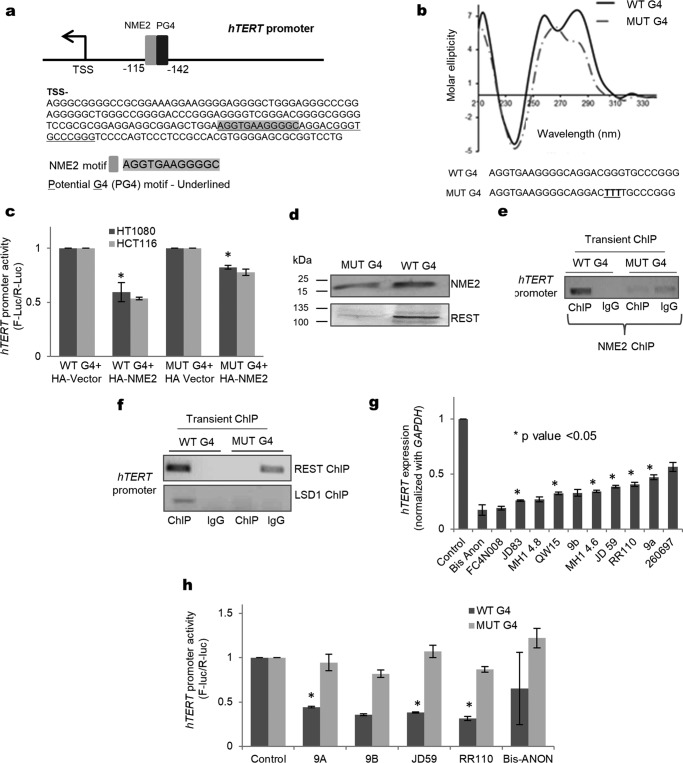
**Repression of *hTERT* depends on NME2 interaction with *hTERT* promoter G-quadruplex.** *a*, minimal promoter of *hTERT* showing the NME2-binding site (*gray solid bar*) and PG4-forming sequence (*underlined*). *b*, circular dichroism plot showing formation of the G4 motif in solution, whereas the sequence with mutations in key bases (MUT-G4) showed partial disruption of the G4 motif under similar conditions. *TSS*, transcription start site. *c*, luciferase reporter assay for *hTERT* promoter activity upon transfection of either the wild-type G4 or the disrupted MUT-G4 plasmid along with HA vector or HA-NME2 in HT1080 and HCT116 cells. *F-luc*, firefly luciferase; *R-luc*, *Renilla* luciferase. *Error bars* represent S.E. (three biological replicates); * indicates *p* < 0.05. *d*, oligonucleotide pulldown assay with either the WT G4 or the disrupted MUT-G4 oligonucleotide in HT1080 nuclear lysate developed with the respective antibodies shows that both NME2 and REST have enhanced affinity for the G4 motif relative to MUT-G4. *e* and *f*, intracellular NME2 (and REST–LSD1) occupancy at the *hTERT* promoter and the presence of intact/deformed promoter G4 motif. ChIP using plasmid vectors harboring the *hTERT* promoter (*Transient ChIP*) shows that NME2 (*e*) and REST–LSD1 (*f*) occupancy at the *hTERT* promoter was significantly lost in the case of the plasmid with the disrupted G4 motif (MUT G4) relative to the plasmid with the WT G4 motif in HT1080 cells. ChIP-PCR was performed using primers specific for the plasmid. *g*, *hTERT* expression in HT1080 cells following treatment with G-quadruplex–binding ligands. *Error bars* represent S.E. (three biological replicates); * indicates *p* < 0.05. *h*, effect of selected G-quadruplex–binding ligands on *hTERT* promoter activity in the case of reporter vector with either the intact G4 motif (WT G4) or the disrupted G4 motif (MUT-G4) in HT1080 cells. *Error bars* represent S.E. (three biological replicates); * indicates *p* < 0.05.

Potential G4 (PG4)-forming sequences were reported in the *hTERT* promoter (29, 51). Interestingly, we observed that the NME2-binding motif (Fig. 4*a*) was within also within a *hTERT* promoter PG4-forming sequence, and because NME2 and promoter G4 interactions have been reported before (21, 30), we asked whether the G4 structure played any role in *hTERT* repression by NME2. Oligonucleotide representing the PG4-forming sequence adopted mixed parallel/antiparallel G4 structures in solution (Fig. 4*b*). For further work, we designed nucleotide substitutions such that key bases required for G4 stability were mutated, but the NME2-binding motif remained intact. This resulted in a disrupted G4 structure in solution (Fig. 4*b*). Next, a luciferase reporter construct was made by substituting these bases (MUT-G4) using site-directed mutagenesis. Upon expression of NME2, although promoter activity was repressed by ∼50% in the case of the G4-containing construct, for MUT-G4 the observed repression was relatively less in HT1080 and HCT116 cells (Fig. 4*c*).

To test NME2 interaction with the G4, we used oligonucleotide pulldown with the G4 or the MUT-G4 sequence. On probing with anti-NME2 antibody, we found relatively reduced affinity of NME2 toward the MUT-G4 compared with G4 (Fig. 4*d* and supplemental Fig. S4). In addition, we checked and observed that a significantly reduced amount of REST was pulled down in the case of MUT-G4, supporting NME2–REST interaction (Fig. 4*d*). Furthermore, we found reduced intracellular occupancy of NME2 at the *hTERT* promoter in the case of ectopically maintained MUT-G4 relative to G4 in ChIP with anti-NME2 antibody followed by PCR with plasmid-specific primers (Fig. 4*e*; also called transient ChIP (31)). This was also confirmed by ChIP using anti-REST or anti-LSD1 where reduced occupancy was observed for both REST and LSD1 on the *hTERT* promoter in the case of MUT-G4 (Fig. 4*f*). Together, these results support roles of the G4 structural motif in NME2 binding and recruitment of the REST–LSD1 complex for *hTERT* repression.

We next studied the effect of G4-binding ligands on telomerase transcription. Because G4 ligands were noted to both activate and repress gene transcription (32, 52), we screened 20 previously established intracellular G4-binding ligands (listed in supplemental Table S2). Of these, 11 ligands showed down-regulation (∼50–85%) of *hTERT* in HT1080 cells (Fig. 4*g* and supplemental Table S2). Five of the 11 ligands also showed more than 50% reduction in *hTERT* promoter activity in reporter assays (Fig. 4*h*), and the ligand-induced effect on promoter activity was lost in the case of all five ligands when the G4 motif was mutated in the reporter construct. This further supported a role of G4–ligand interactions inside cells in *hTERT* repression (Fig. 4*h*).

## Discussion

Multiple transcription factors including Sp1, c-Myc, p53, SP1, ETS, E2F, and AP1 have been reported to transcriptionally control *hTERT* expression (10, 33, 34). However, how these function to control the epigenetic state of the *hTERT* promoter is not precisely understood. For example, histone deacetylation repressed *hTERT* transcription, whereas treatment with the HDAC inhibitor trichostatin A resulted in activation of *hTERT* (35), and a recent report suggested that LSD1, using its demethylation activity, altered activating histone marks on the *hTERT* promoter to repress *hTERT* transcription (36). Although these studies indicated the participating regulatory factors, mechanisms of how the repressors/histone modifiers were engaged on the *hTERT* promoter have remained unclear.

Herein we show that NME2 transcriptionally represses *hTERT* through recruitment of the REST repressor complex, which includes REST, HDAC1, HDAC2, and LSD1. Using direct and sequential ChIP, we found that association of the REST repressor complex on the *hTERT* promoter was dependent on NME2. Moreover, co-immunoprecipitation experiments confirmed that NME2 interacted with members of the REST complex inside the nucleus. As a result, we observed that the *hTERT* promoter is maintained in a repressive chromatin state. In other words, these data suggest that the loss of NME2 results in permissive chromatin at the *hTERT* promoter and up-regulation of *hTERT* expression.

Low NME2 has been associated with malignant transformation in multiple cancers (14, 18). Independently, in oral, breast, and colon cancer, mechanisms of antimetastatic action of NME2 were suggested to be through induction of the epithelial phenotype (mesenchymal to epithelial transition) (37). Conversely, increased telomerase due to telomerase reactivation was noted in progression of various cancers including acute leukemia, breast, prostate, lung, and melanoma (38). Recent studies further reveal that point mutations in the *hTERT* promoter leading to increased *hTERT* mRNA synthesis is key to *hTERT* reactivation in several cancers (10, 39). In this context, possible implications of low NME2 in up-regulation of *hTERT* transcription through epigenetic alteration of the *hTERT* promoter could be of interest in understanding mechanisms of telomerase activation. Although results suggest engagement of the repressor complex in an NME2-dependent manner, further studies will be required to understand how other transcription factors at the *hTERT* promoter influence this mechanism. NME2 was also noted to inhibit telomerase catalytic activity *in vitro* (40). Together with findings reported here, this indicates a possible dual role of NME2 in control of telomerase in cancer cells.

Multiple lines of evidence support a role of G4 motifs in gene expression. Prominent among these are enrichment of potential G4 forms within promoters across species (41–43) and evidence supporting their role in gene expression (44) including their presence *in vivo* and identification of proteins that function in association with G4s inside cells (45–48). Interestingly, NME2 was reported to regulate transcription through interaction with a c-*myc* promoter G4 motif (21). Together, these data prompted a closer look when we noted that the *hTERT* promoter harbors G4-forming sequences that were implicated in possible *hTERT* expression (29). Our results support a role of *hTERT* promoter G4 motifs in hTERT repression. This encouraged us to test G4 ligand-mediated repression of *hTERT* expression, which in turn may open potential ways of controlling *hTERT* activation in cancer cells.

## Experimental procedures

### Cells and culture conditions

HT1080, HCT116, and MRC5 primary fibroblast cells were obtained from the American Type Cell Culture (ATCC) and maintained in modified Eagle's medium (MEM), Dulbecco's modified Eagle's medium low glucose (DMEM) with high glucose, and MEM, respectively, and supplemented with 10% fetal bovine serum at 37 °C in 5% CO_2_.

### ChIP

ChIP assays were performed as described (40) with the following antibodies: rabbit anti-HA antibody (Abcam, ab9110), anti-rabbit IgG (Sigma), anti-REST antibody (Millipore, 17641), anti-LSD1 (Abcam, ab17721), anti-HDAC1 (Abcam, ab7028), anti-HDAC2 (Abcam, ab51832), anti-H3K4me (Abcam, ab8895), anti-H3K4me2 (Abcam, ab7766), anti-H3K4me3 (Abcam, ab8580), anti-histone3 (Abcam, ab1791), and anti-H3k9ac (Abcam, ab10812). For sequential ChIP, samples were treated as described for ChIP (40) before adding 3–4 μg of the first antibody and incubating overnight at 4 °C followed by 50 μl of protein G-Sepharose beads for 4 h at 4 °C on a rotator. Samples were then washed with washing buffers (low-salt buffers, high-salt buffers, and lithium chloride) and divided into two fractions. One part was processed further for ChIP-DNA elution, and 3–4 μg of the second antibody was added to the second fraction and incubated again overnight at 4 °C followed by incubation with 50 μl of protein G-Sepharose beads. Beads were washed (washing buffers as above), and the final DNA samples were obtained using a phenol, chloroform, and isoamyl alcohol precipitation method (as for basic ChIP protocol). The ChIP PCR primers used for *hTERT* promoter were 5′-CCAGGCCGGGCTCCCAGTGGAT-3′ (forward) and 5′-GGCTTCCCACGTGCGCAGCAGGA-3′ (reverse), and the amplicon length was 275 bp. For transient ChIP assays, cells were transfected with wild-type *hTERT* and mutant *hTERT* promoter constructs (2 μg of plasmid/1 × 10^6^cells). Transfected cells were harvested after 48 h, and ChIP was performed against REST, NME2, and LSD1 as described above. The eluted ChIP-DNA was checked for *hTERT* promoter amplification by using primers derived partially from the vector construct and from the *hTERT* promoter sequence.

### Real-time PCR

Total RNA was isolated using TRIzol® reagent (Invitrogen, Life Technologies) according to the manufacturer's instructions. RNA was quantified, and 2 μg of RNA was used for cDNA preparation using an Applied Biosciences kit. The relative transcript expression level for genes was measured by quantitative real-time PCR using a SYBR Green-based method. Average -fold change was calculated by the difference in threshold cycles (Ct) between test and control samples. *GAPDH* gene was used as an internal control for normalizing the cDNA concentration of each sample. For TCP (concentration, 1 μm)-treated cells, treatment was done after 24 h of transfection, and RNA was isolated 24 h post-treatment. G-quadruplex ligand (concentration, 2 μm) treatment was done for 48 h.

### siRNA transfection

SMARTpool ON-TARGET siRNAs against NME2, NME1, and REST were procured from GE Dharmacon and used according to the manufacturer's protocol.

### Luciferase assay

The minimal promoter region of *hTERT* harboring the NME2 motif and G-quadruplex sequences was cloned into pGL3-basic vector and transfected into HT1080 and HCT116 cells using Lipofectamine 2000 (Invitrogen). Plasmid (pGL4.73) containing a CMV promoter driving *Renilla* luciferase was co-transfected as a transfection control for normalization. After 48 h, cells were harvested, and luciferase activities of cell lysate were measured using a Dual-Luciferase reporter assay kit (Promega). For TCP (concentration, 1 μm)-treated cells, treatment was done after 24 h of transfection, and luciferase activity was measured 24 h post-treatment. G-quadruplex ligand (concentration, 2 μm) treatment was done for 48 h.

### Preparation of nuclear extracts

HT1080 cells grown in MEM supplemented with 10% FBS (Sigma) were collected and washed in cold 1× PBS, and nuclear extract was isolated using a nuclear extraction kit (CelLytic, Sigma) according to the manufacturer's protocol.

### Co-IP

For immunoprecipitation experiments, 500 μg of nuclear extract was incubated for 4 h at 4 °C with 4 μg of anti-NME2 antibody (Kamiya, KM1121 MC412). Immunoprecipitation was performed using a Catch and Release co-immunoprecipitation kit (Millipore) according to the manufacturer's protocol.

### Antibodies and Western blotting

For Western analysis, immunoprecipitated nuclear extracts were separated by 10% sodium dodecyl sulfate (SDS)-PAGE and transferred to polyvinylidene difluoride (PVDF) membranes (Immobilon FL, Millipore). The following primary antibodies were used for immunoblotting: anti-REST antibody (Millipore, 17–641), anti-HDAC1 (Abcam, ab7028), anti-HDAC2 (Abcam, ab51832), anti-LSD1 (Abcam, ab17721), anti-NME2 (Abcam, ab60602) (23, 24, 40), and anti-hTERT (Abcam, ab32020) (49, 50). The secondary antibodies used were anti-mouse and anti-rabbit alkaline phosphatase conjugates from Sigma. Western blots with antibodies against NME2, telomerase, REST, and LSD1 along with relevant molecular weight markers are shown in supplemental Fig. S5.

### Immunofluorescence microscopy

Cells were grown on coverslips and at 100% confluence were fixed with 4% paraformaldehyde by incubating for 10 min at room temperature. Cells were permeabilized with 0.5% Triton^TM^ X-100 and treated with blocking solution (3% BSA in PBS) for 30 min at room temperature. After one PBS (1×) wash, cells were treated with anti-hTERT antibody (1:100) overnight at 4 °C. The next day cells were washed alternately with 1× PBS and PBS with Tween 20 three times and probed with Alexa Fluor® 594 for 1 h at room temperature. Cells were washed again alternately with PBS and PBS with Tween 20 three times and mounted with Prolong® Gold antifade reagent with DAPI. Images were taken as maximum intensity projections on a Leica TCS-SP8 confocal microscope.

### Flow cytometry (FACS)

After trypsinization, both stable GFP-NME2-expressing and corresponding GFP vector-transformed cells were fixed in 4% paraformaldehyde for 10 min. After washing in PBS, cells were permeabilized in 0.5% Triton X-100 in PBS for 10 min. After blocking in 3% BSA in PBS for 1 h at 4 °C followed by washing in PBS, cells were incubated with primary anti-hTERT antibody (Abcam, ab94523) (1:100) at 4 °C overnight. After washing in PBS, cells were incubated with Alexa Fluor 594-conjugated secondary antibody for 2 h at 4 °C. As a negative control, cells were fixed and incubated with secondary antibody only without incubation with primary antibody. Finally, after washing in PBS, cells were resuspended in PBS for acquisition by flow cytometry (BD Biosciences FACSAria III) using the Cy3 channel (for Alexa Fluor 594) to detect *hTERT* expression.

### Oligonucleotide pulldown assay

For pulldown assays, 2 μg of biotinylated NME2 motif, motif mutant M1, wild-type G4, and MUT-G4 and 300 μg of nuclear extract (prepared from HT1080 cells) were incubated for 1 h at room temperature in binding buffer. Then the whole complex was incubated with 60 μl of streptavidin-agarose (Invitrogen) for 4 h at 4 °C. In all experiments, the beads were washed three times with washing buffer (50 mm Tris-HCl (pH 7.5) and 150 mm NaCl), and the bound proteins were eluted by boiling in 5× SDS sample buffer (20 mm Tris-HCl (pH 6.8), 10% glycerol, 4% SDS, 100 mm dithiothreitol, 4 mm EDTA, and 0.025% Coomassie Brilliant Blue R-250) and subjected to Western blot analysis using anti-REST antibody (Abcam) and anti-NME2 antibody (Kamiya).

### Separation of immunoprecipitate and preparation of peptide mixtures

Protein identification using mass spectrometry was performed as follows. First, the immunoprecipitate was resolved by 12.5% SDS-PAGE followed by visualization with colloidal Coomassie Brilliant Blue. The bands that appeared specifically in the immunoprecipitate were excised manually using sterile blades. Further procedures including distaining and digestion with trypsin followed by extraction of peptides were performed manually. In brief, gel pieces were distained in 50 mm ammonium bicarbonate and acetonitrile (50%, v/v) and dehydrated with 95% (v/v) acetonitrile. The dehydrated gel pieces were soaked with a sufficient volume of trypsin solution (20 ng/μl trypsin in 25 mm ammonium bicarbonate) and incubated at 37 °C overnight. Upon digestion, the trypsinized solution was pooled with a further peptide extraction from gel pieces using 50% (v/v) acetonitrile containing 0.1% (v/v) acetic acid and incubating for 30 min at 37 °C with constant shaking. The peptide extract collected as supernatant was pooled, vacuum-dried to about 10 μl, and purified using a micro ZipTip (Millipore). The purified mixture was vacuum-dried, and peptides were reconstituted in 2% acetonitrile containing 0.1% (v/v) acetic acid and further analyzed by LC-MS/MS.

### Identification of proteins using LC-MS/MS

Protein identification from peptide mixtures was performed on an LTQ Orbitrap Velos mass spectrometer (Thermo Scientific, Germany) equipped with a nanoelectrospray ion source coupled with an on-line Proxeon Easy nLC (Thermo Scientific). Briefly, the peptides were separated using an Acclaim PepMap 100 analytical column (C18; particle size, 3 μm; 75-μm inner diameter; 15 cm long; 100 Å; LC Packings, Germany) at a flow rate of 300 nl/min. Before separation, the peptide mixtures were enriched using an NS-MP-10 Biosphere precolumn (C18; particle size, 5 μm; 100-μm inner diameter; 20 mm long; pore size, 120 Å; Nano Separations, Netherlands). The peptides were eluted with a mixture of solvent gradient (2–5% buffer B in 1 min, 5–25% buffer B in 59 min, 25–40% buffer B in 10 min, and 100% buffer B in 8 min) of buffer A (2% acetonitrile containing 0.1% acetic acid) and buffer B (acetonitrile containing 0.1% acetic acid). The eluted peptides were electrosprayed into the mass spectrometer. The mass spectrometric data were collected in data-dependent mode to switch between Orbitrap MS and LTQ MS/MS acquisition automatically. Full-scan MS spectra in the *m*/*z* range from 300 to 1700 (resolution *r* = 30,000) were acquired in the Orbitrap mass spectrometer. The data acquisition method was set to isolate up to 20 of the most intense ions depending upon signal intensity for fragmentation in the linear ion trap using collision-induced dissociation. Target ions already selected for MS/MS were dynamically excluded for 60 s. The general conditions of the mass spectrometer for data collection were 1.6–1.7-kV electrospray voltage and ion selection threshold of 2000 counts for MS/MS. For identification of proteins from MS data, an automated database search was performed using Proteome Discoverer 1.3.0.339 (Thermo Scientific) with the Sequest algorithm. A human UniProt FASTA database and a decoy database of 1% false discovery rate were used for identification. Two possible missed cleavages for trypsin enzyme specificity with a mass tolerance of 10 ppm (parent ion) and 0.8 Da (fragment ion) and methionine oxidation dynamics were considered for the database search. The identity of the proteins was confirmed based on high-confidence peptide identification containing at least two peptides per protein (with rank 1 peptides in proteins; XCorr score, ≥2.45 or 2.85 for doubly and triply charged peptides, respectively).

### Site-directed mutagenesis

A QuikChange site-directed mutagenesis kit (Agilent technologies) was used to generate various mutants of *NME2* gene. Mutagenesis reactions were performed according to the manufacturer's instructions with the wild-type c-DNA cloned in pRSET-A used as a template. The positive clones were screened for the mutation by sequencing.

## Author contributions

D. S. and S. C. designed the experiments. T. H. and D. S. performed immunofluorescence experiments. D. S. and A. S. performed ChIP-seq. D. S., P. D., and A. K. performed ChIP-seq validation. A. S. performed the biophysical experiments. S. S. and T. H. performed flow cytometry experiments. T. H. and D. S. performed ligand screening. V. D., R. U., and D. S. performed proteomics experiments. V. S. performed the telomere repeat amplification protocol. D. S., T. H., and S. C. contributed to data compilation, images, and manuscript writing. S. C. conceived the idea, conceptualized the study, wrote and edited the manuscript, and is responsible for correspondence.

## Supplementary Material

Supplemental Data

## Footnotes

7 The abbreviations used are:

hTERT: human telomerase reverse transcriptase
REST: RE1-silencing transcription factor
LSD1: lysine-specific histone demethylase 1
NME2: non-metastatic 2
G4: G-quadruplex
MUT: mutated
co-IP: co-immunoprecipitation
TCP: tranylcypromine
PG4: potential G4
HDAC: histone deacetylase
MEM: modified Eagle's medium.

## Note added in proof

An incorrect gel section was inadvertently used in the REST ChIP panel in Fig. 3*f* of the version of this article that was published as a Paper in Press on July 17, 2017. This error has now been corrected and does not affect the results or conclusions of this work.
