# Supplementary material for: Epigenetic suppression of human telomerase (hTERT) is mediated by the metastasis suppressor NME2 in a G-quadruplex–dependent fashion
Source: J Biol Chem. 2017 Jul 17;292(37):15205–15. doi: 10.1074/jbc.M117.792077 (PMC5602382; doi:10.1074/jbc.M117.792077)
Supplement: Supplemental Data [file supp_292_37_15205__index.html]

Epigenetic suppression of human telomerase (hTERT) is mediated by the metastasis suppressor NME2 in a G-quadruplex–dependent fashion — Epigenetic regulation of hTERT — Supplemental Data 

# Epigenetic suppression of human telomerase (*hTERT*) is mediated by the metastasis suppressor NME2 in a G-quadruplex–dependent fashion

## Supplemental Data

- Supplementary Figures and Tables
