# Supplementary material for: Epigenetic suppression of human telomerase (hTERT) is mediated by the metastasis suppressor NME2 in a G-quadruplex–dependent fashion
Source: J Biol Chem. 2017 Jul 17;292(37):15205–15. doi: 10.1074/jbc.M117.792077 (PMC5602382; doi:10.1074/jbc.M117.792077)
Supplement: Supplemental Data [file supp_M117.792077_supp_data.pdf]

Supplementary Fig S1

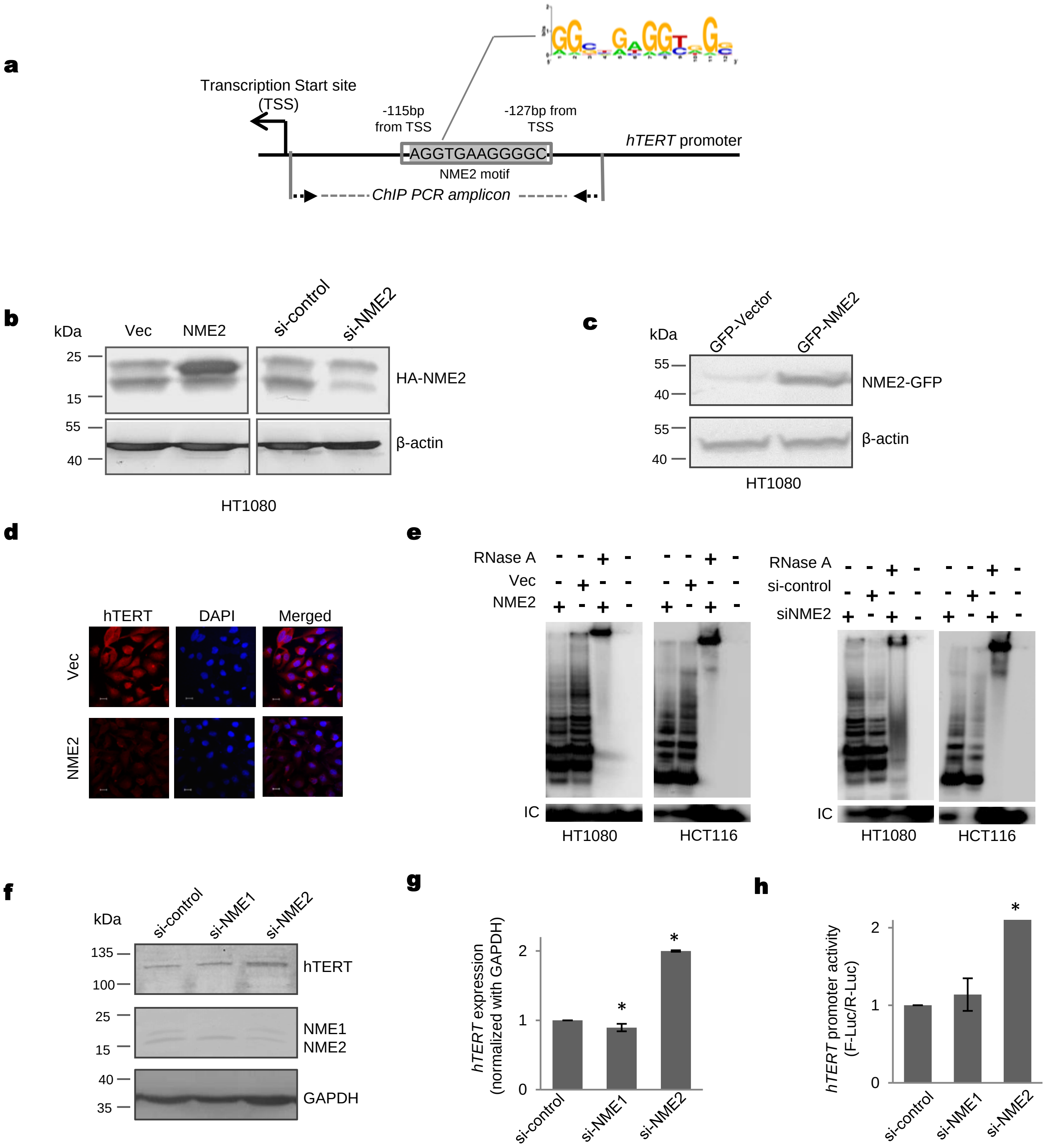

Figure S1: (a) *hTERT* promoter showing NME2 motif obtained from ChIP-Seq (Ref.24). ChIP-PCR primers were designed for *hTERT* promoter marked with black arrow; **(b)** Western blot showing over-expression and silencing of NME2 in HT1080 and HCT116 cell lines. **(c)** Protein expression of NME2 in NME2 stable over-expressed (GFP-NME2) HT1080 cells. **(d)** Immunofluorescence shows reduced expression of telomerase (in red) in GFP-NME2 stable over-expressed cells in comparison with GFP Vector HT1080 cells. Middle panel shows DAPI. **(e)** TRAP results showing reduced and increased telomerase activity in HT1080 and HCT116 cell lines in NME2 over-expressed and silenced conditions respectively. **(f)** Western blot showing *hTERT* expression after silencing of NME1and NME2 in HT1080 cells. **(g-h)** Real time PCR for *hTERT* expression (f) and luciferase reporter assay for *hTERT* promoter (g) in NME1 silenced condition in HT1080 cells. Error bar represents SE (three biological replicates); ***** indicates p value <0.05.

Supplementary Fig S2

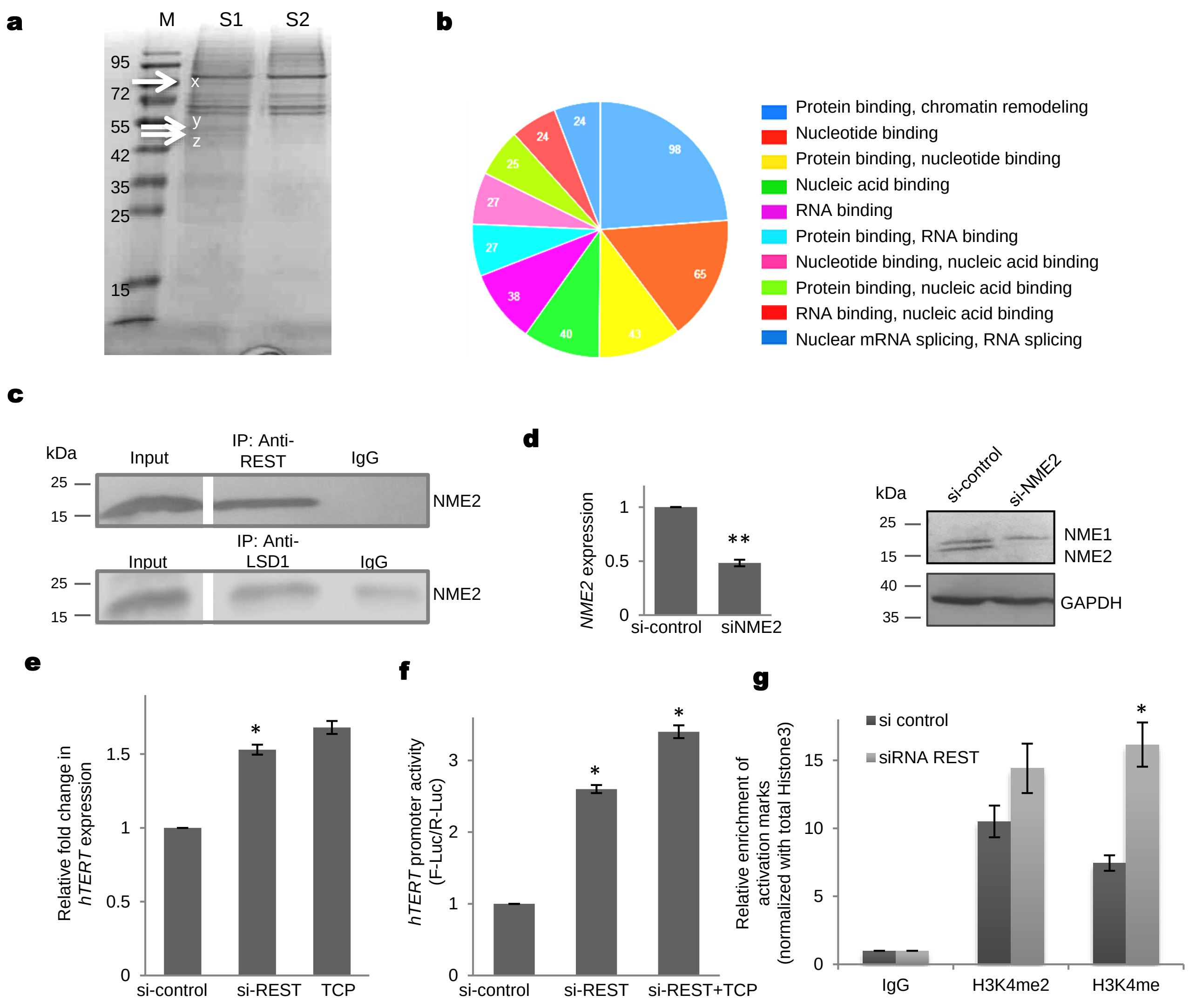

Figure S2: (a) Gel image showing samples (S1, S2) used in LC MS/MS analysis. Arrows indicate bands (x,y,z) which were used for LC- MS/MS analysis. **(b)** Gene ontology analysis showing proteins obtained from LC-MS/MS analysis, belong to different classes. **(c)** Reverse Co-Immunoprecipitation blots showing interacting of REST with NME2 (upper panel) and LSD1 with NME2 (lower panel). **(d)** mRNA and protein expression analysis of NME2 in HT1080 cells. Error bar represents SE (three biological replicates); ** indicates p value <0.005. **(e)** Expression of hTERT was checked in silencing of REST and blocking of LSD1 and HDAC1/2 complex independently in HT1080 cells. Error bar represents SE (three biological replicates); * indicates p value <0.05. **(f)** Luciferase assay shows *hTERT* promoter activity in REST silenced condition and blocking of LSD1 (using TCP -1uM conc.) in combination. Error bar represents SE (three biological replicates); * indicates p value <0.05. **(g)** ChIP results show relatively increased activation histone marks (H3K4me2 and H3K4me) at *hTERT* promoter; ChIP normalized by total Hisotne 3 (H3) ChIP; IgG used as negative control. Error bar represents SE (three biological replicates). Error bar represents SE (three biological replicates); * indicates p value <0.05.

Supplementary Figure S3

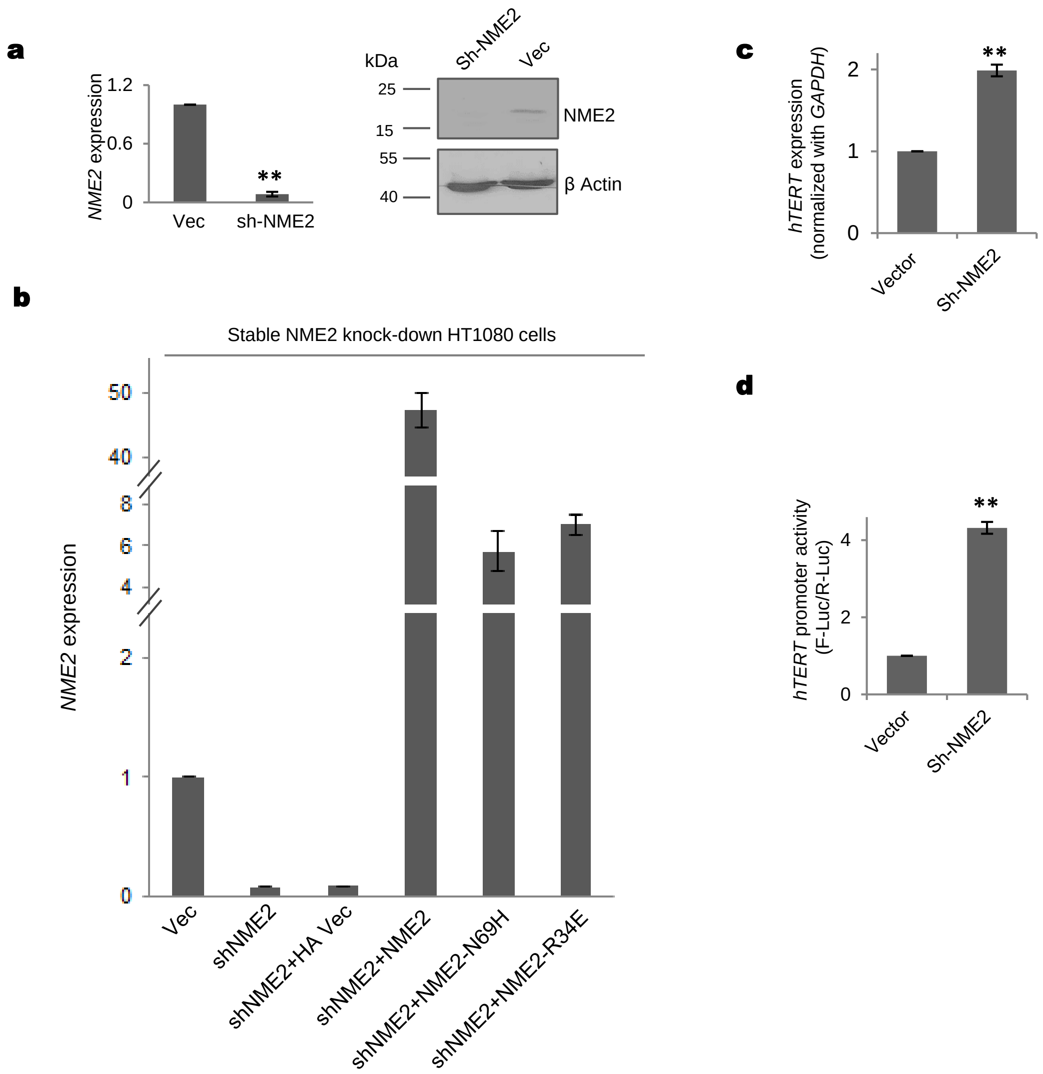

Figure S3- (a) mRNA and western blot showing NME2 expression in HT1080-shNME2 cells Error bar represents SE (three biological replicates); ****** indicates p value <0.005. **(b)** NME2 expression in stable NME2 knockdown shNME2 HT1080 cells after transfected with different NME2 mutants. Error bar represents SE (three biological replicates); **(c-d)** *hTERT* expression (c) and promoter activity (d) measured in stable NME2 knockdown HT1080 cells. Error bar represents SE (three biological replicates); ****** indicates p value <0.005.

Supplementary Fig S4

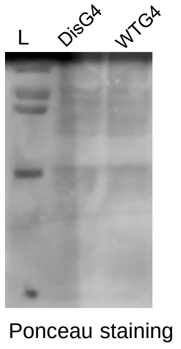

Figure S4: Ponceau staining shows equal loading of lysates in oligonucleotide pull down assay

Supplementary Figure S5

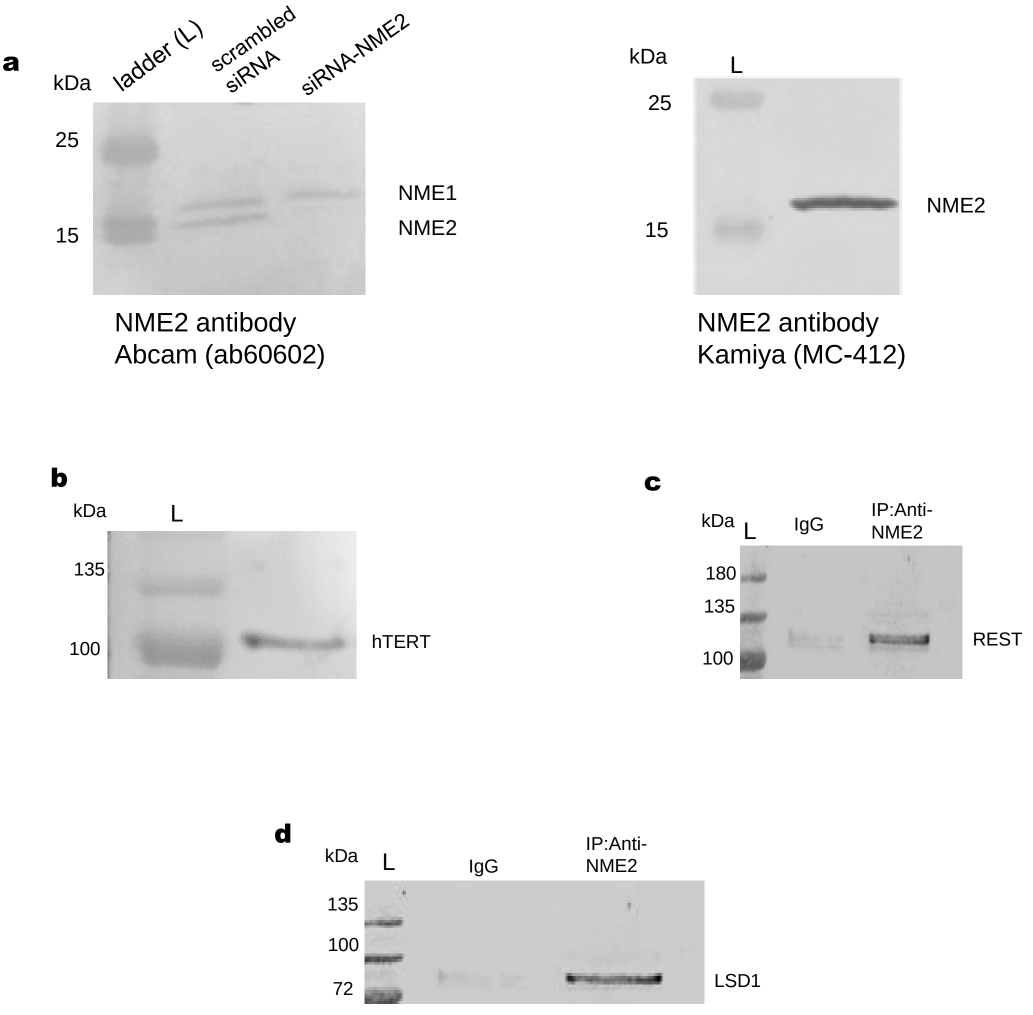

Figure S5: (a) Western blot showing NME1 / NME2 expression; NME2 band was lost on treatment with siRNA specific for NME2 (control scrambled vs siRNA NME2) using Abcam ab60602 antibody (left panel). Right panel shows NME2 western blot using NME2-specific antibody from Kamiya MC-412 **(b)** Western blot showing hTERT expression using Abcam ab32020 antibody. **(c-d)** Western blots showing REST (c) and LSD1 (d) using antibody from Millipore (17641) and Abcam (ab17721) respectively.

Supplementary Table S1- List of proteins found in LC-MS/MS experiments

Query Ref	Gene symbol	Gene name	Biological Functions
F5H0N0	ACTG1	actin, gamma 1	nucleotide binding; protein binding
D6PXK4	ACTN4	actinin, alpha 4	response to hypoxia; protein binding
F5H3P5	ACTR3	ARP3 actin-related protein 3 homolog (yeast)	Golgi membrane; protein binding
Q8TA92	AFG3L2	AFG3 ATPase family gene 3-like 2 (S. cerevisiae)	nucleotide binding
F5H737	AHCY	adenosylhomocysteinase	adenosylhomocysteinase activity
Q8N274	AHNAK	AHNAK nucleoprotein	nucleic acid binding; protein binding
E7ESU5	ALB	albumin	platelet degranulation; protein binding
Q96SM7	ARID1A	AT rich interactive domain 1A (SWI-like)	nuclear chromatin; protein binding
B7Z3U6	ATP1A1	ATPase, Na+/K+ transporting, alpha 1 polypeptide	nucleotide binding
A8K092	ATP5A1	ATP synthase, H+ transporting, mitochondrial F1 complex, alpha subunit 1, cardiac muscle	nucleotide binding; protein binding
E9PGE6	ATP6V1H	ATPase, H+ transporting, lysosomal 50/57kDa, V1 subunit H	vacuolar proton-transporting V-type ATPase, V1 domain; protein binding
E7ESH8	ATXN10	ataxin 10	protein binding
Q96G95	BCKDK	branched chain ketoacid dehydrogenase kinase	two-component sensor activity
E9PK09	BCLAF1	BCL2-associated transcription factor 1	DNA binding; protein binding
E7ESP5	BRD4	bromodomain containing 4	regulation of transcription involved in G1 phase of mitotic cell cycle
F5H879	CALU	calumenin	platelet degranulation; protein binding
B4E2T8	CANX	calnexin	antigen processing and presentation of peptide antigen via MHC class I; protein binding
A8MVQ3	CARS	cysteinyl-tRNA synthetase	tRNA binding; protein binding
F8VQ14	CCT2	chaperonin containing TCP1, subunit 2 (beta)	nucleotide binding
B4DYD8	CCT5	chaperonin containing TCP1, subunit 5 (epsilon)	nucleotide binding; protein binding
Q5QPR4	CDK11A	cyclin-dependent kinase 11A	nucleotide binding
F5H3Y6	CEBPZ	CCAAT/enhancer binding protein (C/EBP), zeta	DNA binding
F8WJN3	CPSF6	cleavage and polyadenylation specific factor 6, 68kDa	nucleotide binding; protein binding
F5H669	CPSF7	cleavage and polyadenylation specific factor 7, 59kDa	nucleotide binding; protein binding
B4DJV2	CS	citrate synthase	citrate (Si)-synthase activity
E9PGZ0	CSDE1	cold shock domain containing E1, RNA-binding	nucleic acid binding
A3KFI9	CSTF1	cleavage stimulation factor, 3' pre-RNA, subunit 1, 50kDa	nuclear mRNA splicing, via spliceosome; protein binding
F5H0G6	CSTF3	cleavage stimulation factor, 3' pre-RNA, subunit 3, 77kDa	nuclear mRNA splicing, via spliceosome
B3KTW0	CUL1	cullin 1	G1/S transition of mitotic cell cycle
A6NE76	CUL4B	cullin 4B	protein binding
Q68CR9	DARS	aspartyl-tRNA synthetase	nucleotide binding
F5H1F9	DBT	dihydrolipoamide branched chain transacylase E2	cytoplasm

Query Ref	Gene symbol	Gene name	Biological Functions
F8WAG8	DCTN2	dynactin 2 (p50)	G2/M transition of mitotic cell cycle; protein binding
E7EWT1	DDOST	dolichyl-diphosphooligosaccharide--protein glycosyltransferase	dolichyl-diphosphooligosaccharide-protein glycotransferase activity
B4DPN6	DDX1	DEAD (Asp-Glu-Ala-Asp) box polypeptide 1	nucleotide binding
C9JMU5	DDX17	DEAD (Asp-Glu-Ala-Asp) box polypeptide 17	nucleotide binding
F5H863	DDX18	DEAD (Asp-Glu-Ala-Asp) box polypeptide 18	nucleotide binding
F5H4J5	DDX24	DEAD (Asp-Glu-Ala-Asp) box polypeptide 24	nucleotide binding
Q3MI07	DDX27	DEAD (Asp-Glu-Ala-Asp) box polypeptide 27	nucleotide binding
F5H3H6	DDX50	DEAD (Asp-Glu-Ala-Asp) box polypeptide 50	nucleotide binding
A8MTP9	DDX52	DEAD (Asp-Glu-Ala-Asp) box polypeptide 52	nucleotide binding
F5H6K0	DHX15	DEAH (Asp-Glu-Ala-His) box polypeptide 15	nucleotide binding
F5GXA5	DHX9	DEAH (Asp-Glu-Ala-His) box polypeptide 9	nucleotide binding
B7Z5W8	DLST	dihydrolipoamide S-succinyltransferase (E2 component of 2-oxo-glutarate complex)	dihydrolipoyllysine-residue succinyltransferase activity
F8W884	DNAJC10	DnaJ (Hsp40) homolog, subfamily C, member 10	ATPase activator activity; protein binding
Q96NI0	DOCK7	dedicator of cytokinesis 7	guanyl-nucleotide exchange factor activity
D3YTH2	DOCK9	dedicator of cytokinesis 9	guanyl-nucleotide exchange factor activity; protein binding
B4DT58	DPF2	D4, zinc and double PHD fingers family 2	nucleic acid binding; protein binding
Q6IQ15	EEF1A1	eukaryotic translation elongation factor 1 alpha 1	nucleotide binding; protein binding
B4DTG2	EEF1G	eukaryotic translation elongation factor 1 gamma	translation elongation factor activity; protein binding
B4DK30	EFTUD2	elongation factor Tu GTP binding domain containing 2	nucleotide binding; protein binding
E7EQG2	EIF4A2	eukaryotic translation initiation factor 4A2	nucleotide binding
F5H1C3	ENO2	enolase 2 (gamma, neuronal)	phosphopyruvate hydratase complex
Q96CG1	ETF1	eukaryotic translation termination factor 1	nuclear-transcribed mRNA catabolic process, nonsense-mediated decay; protein binding
B4DKG8	EXOSC10	exosome component 10	nucleotide binding
E9PH82	FAM98A	family with sequence similarity 98, member A	
A8MUW5	FAM98B	family with sequence similarity 98, member B	tRNA-splicing ligase complex
F5H1Y3	FEN1	flap structure-specific endonuclease 1	S phase of mitotic cell cycle
E7EWU2	FLNA	filamin A, alpha	glycoprotein binding
F8WAN4	FRMD4A	FERM domain containing 4A	binding
E7EU23	GDI2	GDP dissociation inhibitor 2	Rab GDP-dissociation inhibitor activity; protein binding
F5GYQ4	GLUD1	glutamate dehydrogenase 1	nucleotide binding
A6NI00	GNAS	GNAS complex locus	nucleotide binding

Query Ref	Gene symbol	Gene name	Biological Functions
F5H5I6	GRSF1	G-rich RNA sequence binding factor 1	nucleotide binding
B4DTJ5	GTF2H4	general transcription factor IIH, polypeptide 4, 52kDa	nucleotide-excision repair, DNA damage removal; protein binding
B7Z7A3	GTPBP4	GTP binding protein 4	regulation of cyclin-dependent protein kinase activity; protein binding
B4E2W0	HADHB	hydroxyacyl-CoA dehydrogenase/3-ketoacyl-CoA thiolase/enoyl-CoA hydratase (trifunctional protein), beta subunit	fatty-acyl-CoA binding
B4DY73	HARS	histidyl-tRNA synthetase	nucleotide binding
F5GXM1	HDAC1	histone deacetylase 1	histone deacetylase complex
B3KRS5	HDAC2	histone deacetylase 2	histone deacetylase complex
B4DG62	HK1	hexokinase 1	nucleotide binding
B4DKS8	HNRNPF	heterogeneous nuclear ribonucleoprotein F	nucleotide binding
E9PCY7	HNRNPH1	heterogeneous nuclear ribonucleoprotein H1 (H)	nucleotide binding; protein binding
B4DFK9	HNRNPH2	heterogeneous nuclear ribonucleoprotein H2 (H')	nucleotide binding
Q5T6W5	HNRNPK	heterogeneous nuclear ribonucleoprotein K	nuclear mRNA splicing, via spliceosome
A6NIT8	HNRNPL	heterogeneous nuclear ribonucleoprotein L	nucleotide binding; protein binding
Q59ES8	HNRNPM	heterogeneous nuclear ribonucleoprotein M	nucleotide binding
E7ERE4	HNRNPR	heterogeneous nuclear ribonucleoprotein R	nucleotide binding; protein binding
B3KX72	HNRNPU	heterogeneous nuclear ribonucleoprotein U (scaffold attachment factor A)	nucleotide binding
B7Z4B8	HNRNPUL1	heterogeneous nuclear ribonucleoprotein U-like 1	nuclear mRNA splicing, via spliceosome
E7EPL9	HSD17B4	hydroxysteroid (17-beta) dehydrogenase 4	very long chain fatty acid metabolic process
Q86U12	HSP90AA1	heat shock protein 90kDa alpha (cytosolic), class A member 1	G2/M transition of mitotic cell cycle; protein binding
Q5T9W8	HSP90AB1	heat shock protein 90kDa alpha (cytosolic), class B member 1	nucleotide binding
Q96GW1	HSP90B1	heat shock protein 90kDa beta (Grp94), member 1	nucleotide binding; protein binding
B4DEF7	HSPA5	heat shock 70kDa protein 5 (glucose-regulated protein, 78kDa)	nucleotide binding; protein binding
E9PKE3	HSPA8	heat shock 70kDa protein 8	nucleotide binding; protein binding
F5H3L8	HSPA9	heat shock 70kDa protein 9 (mortalin)	nucleotide binding; protein binding
F8W810	IDBG-19123	Uncharacterized protein	nucleotide binding
F8W930	IGF2BP2	insulin-like growth factor 2 mRNA binding protein 2	nucleotide binding; protein binding
C9JFV5	ILF3	interleukin enhancer binding factor 3, 90kDa	M phase; protein binding
B7Z1I0	ILK	integrin-linked kinase	nucleotide binding
F5GWP8	JUP	junction plakoglobin	cell morphogenesis
E7ET98	KHDRBS1	KH domain containing, RNA binding, signal transduction associated 1	G2/M transition of mitotic cell cycle; protein binding
E7EP96	KHSRP	KH-type splicing regulatory protein	RNA splicing, via transesterification reactions

Query Ref	Gene symbol	Gene name	Biological Functions
B7ZAV6	KPNB1	karyopherin (importin) beta 1	protein import into nucleus, translocation; protein binding
C9JA77	KRT13	keratin 13	structural molecule activity; protein binding
F8VZY9	KRT18	keratin 18	structural molecule activity; protein binding
C9JM50	KRT19	keratin 19	structural molecule activity; protein binding
E7EU87	KRT5	keratin 5	structural molecule activity; protein binding
E7EUE8	KRT6A	keratin 6A	structural molecule activity
F5H6G5	KRT6B	keratin 6B	structural molecule activity
F5GY66	KRT77	keratin 77	molecular_function
F8VXB4	KRT8	keratin 8	structural molecule activity; protein binding
Q6UYC3	LMNA	lamin A/C	structural molecule activity; protein binding
D6RAQ3	LMNA	lamin A/C	structural molecule activity; protein binding
E9PBF6	LMNB1	lamin B1	structural molecule activity
B8ZZ09	LUC7L	LUC7-like (S. cerevisiae)	protein binding
B7Z500	LUC7L2	LUC7-like 2 (S. cerevisiae)	enzyme binding
C9JTA2	MAD1L1	MAD1 mitotic arrest deficient-like 1 (yeast)	cell cycle checkpoint
A8MXP9	MATR3	matrin 3	nucleotide binding; protein binding
B1AHB1	MCM5	minichromosome maintenance complex component 5	cell cycle checkpoint
F5H6D0	MOGS	mannosyl-oligosaccharide glucosidase	catalytic activity
E7ETN4	MRPS27	mitochondrial ribosomal protein S27	mitochondrion
F5H3R4	MTIF2	mitochondrial translational initiation factor 2	nucleotide binding
F8VRJ2	NAP1L1	nucleosome assembly protein 1-like 1	protein binding; protein binding
E7EX81	NCL	nucleolin	nucleotide binding
B7Z9L2	NDUFS2	NADH dehydrogenase (ubiquinone) Fe-S protein 2, 49kDa (NADH-coenzyme Q reductase)	NADH dehydrogenase activity; protein binding
F5GZ79	NLE1	notchless homolog 1 (Drosophila)	inner cell mass cell differentiation; protein binding

Table S2- List of ligands tested for *hTERT* repression

Sl No.	Ligand Name	Reference
1	MH1-4.6	Hampel et al., 2010, Bioorg Med Chem Lett.
2	MH1-4.8	Hampel et al., 2010, Bioorg Med Chem Lett.
3	9a	Sparapani et al., 2010, J Am Chem Soc.
4	9b	Sparapani et al., 2010, J Am Chem Soc.
5	RR 110	Muller et al., 2012, Org Biomol Chem.
6	FC4N008	Collie et al., 2012, J Am Chem Soc.
7	QW15	Brassart et al., 2007, Mol Pharmacol.
8	JD59	Dash et al., 2008, Chem Commun (Camb).
9	260697	Mailliet et al., 2001, patent
10	Bis-ANON	Granzhan et al., 2010, J Nucleic Acids
11	JD83	Dash et al., 2008, Chem Commun (Camb).
